# Multiblock Discriminant Analysis for Integrative Genomic Study

**DOI:** 10.1155/2015/783592

**Published:** 2015-05-17

**Authors:** Mingon Kang, Dong-Chul Kim, Chunyu Liu, Jean Gao

**Affiliations:** ^1^Department of Computer Science and Engineering, University of Texas at Arlington, Arlington, TX 76019, USA; ^2^Department of Computer Science, University of Texas-Pan American, Edinburg, TX 78539, USA; ^3^Department of Psychiatry, University of Illinois at Chicago, Chicago, IL 66012, USA

## Abstract

Human diseases are abnormal medical conditions in which multiple biological components are complicatedly involved. Nevertheless, most contributions of research have been made with a single type of genetic data such as Single Nucleotide Polymorphism (SNP) or Copy Number Variation (CNV). Furthermore, epigenetic modifications and transcriptional regulations have to be considered to fully exploit the knowledge of the complex human diseases as well as the genomic variants. We call the collection of the multiple heterogeneous data “multiblock data.” In this paper, we propose a novel Multiblock Discriminant Analysis (MultiDA) method that provides a new integrative genomic model for the multiblock analysis and an efficient algorithm for discriminant analysis. The integrative genomic model is built by exploiting the representative genomic data including SNP, CNV, DNA methylation, and gene expression. The efficient algorithm for the discriminant analysis identifies discriminative factors of the multiblock data. The discriminant analysis is essential to discover biomarkers in computational biology. The performance of the proposed MultiDA was assessed by intensive simulation experiments, where the outstanding performance comparing the related methods was reported. As a target application, we applied MultiDA to human brain data of psychiatric disorders. The findings and gene regulatory network derived from the experiment are discussed.

## 1. Introduction

Human diseases involve complex processes that include interactive actions of biological multiple layers such as genetic, epigenetic, and transcriptional regulation. Conducting research based on a single type of biological data produces insufficient results to fully exploit the knowledge of the complex human diseases. The prior research shows that it is essential for the study to be based on a comprehensive consideration of the multiple biological data to grasp an in-depth understanding of the complex mechanisms of the human diseases and the identification of disease markers. The recent advances of high-throughput technologies such as DNA microarray and sequencing technologies efficiently profile various types of genomic data. The genomic data include Single Nucleotide Polymorphism (SNP), Copy Number Variation (CNV), DNA methylation (DM), and gene expression (GE). Integrative genomic analysis of the heterogeneous genomic data plays an important role in profiling a global view of a biological system as well as identifying significant markers of the human diseases.

However, most research has focused solely on investigations of a single type of the genomic data. Genome-Wide Association Studies (GWAS) examine genetic loci which are associated with a trait (e.g., major diseases) using the SNP data [1, 2]. GWAS normally compare the SNP arrays of two groups, disease (case) and normal (control) samples. If a genetic variation on a locus with the disease samples is statistically significant to the controls, the SNP is considered associated with the disease, whereas expression Quantitative Trait Loci (eQTL) studies have been actively done to identify genetic loci that regulate gene expression [3]. Combining the gene microarray data with GWAS not only enables the capture of gene regulatory interactions but also provides insight into the genetic mechanism that regulates gene expression variations. However, both GWAS and eQTL mapping studies still remain as a “*missing heritability*” problem [4].

In addition to SNP, Copy Number Variation (CNV) and DNA methylation (DM) have also been highlighted as key factors that affect the gene expression regulation. CNV is a structural alternation of DNA in which specific regions of the genome are deleted or duplicated on chromosomes. Although CNV is frequently observed even in healthy individuals, it is hypothesized that the variants may cause diseases by directly affecting gene dosage and gene expression [5, 6]. Specifically, whole-genome association studies of the relationship between CNV and diseases reported that gene expression levels in CNV regions are strongly related to the deletion or duplication of the regions [6]. Typically, the deletion of either particular regions within a gene or regulatory regions of a gene may result in a lower gene expression than what is normally expressed. DM is an epigenetic modification that occurred by the addition of methyl group to the cytosine or adenine of DNA. DM inhibits transcription of the genes with high levels of 5-methylcytosine in their promoter region or recruits proteins such as histone deacetylases that can modify histones [7, 8]. The functionality of DM consequently changes the gene expression levels even on the same DNA bases.

Thus, recent research has actively extended GWAS and eQTL mapping studies to the integrative association studies with multiple types of genomic data. Most integrative genomic research focuses on identifying genetic, epigenetic, or posttranscriptional factors that control gene expression regulation (or microRNA) by considering the complex interactions of SNP, CNV, and DM [9–11]. Specifically, the Cancer Genomic Atlas [9] conducted large-scale multidimensional analysis with SNP, CNV, DM, and GE to provide comprehensive genomic characterizations for brain cancer. In Aure et al.'s work [10], the combination effects of CNV and DM were examined to identify the association with alterations of miRNA expression in breast tumors. Wagner et al. [11] studied the relationship between SNP, DM, and GE via multiple eQTL analysis.

Most of the integration approaches have used step-by-step processes. Ordinarily, approaches filter candidate markers by using statistical techniques at the first step and find the final markers that satisfy certain criteria at the remaining stages [12–15]. This type of integration method often makes increased “*type II errors*” at each step, that is, fails to find informative markers by incorrectly identifying them as insignificant. Moreover, they do not consider interaction effects of the multiblock data. Mechanism was not considered.

Hence, research has recently started to shift toward approaches using systematical models in order to integrate and analyze the heterogeneous data comprehensively rather than through simple step-wise processes [16–18]. Multiblock methods of Partial Least Squares (PLS) and Generalized Canonical Correlation Analysis (GCCA) are representative methods. A derivative of a sparse version of PLS was proposed by penalizing both features and sample dimensions to identify “*regulatory modules*” [16]. Such PLS-based methods, which maximize the covariance between latent variables, often fail to detect significant factors when their intensities are weak. Furthermore, the method lacks the consideration of the discriminant analysis of the disease. A sparse multiblock analysis method derived from Generalized Canonical Correlation (SGCCA) was developed to identify multiblock association models while considering the relationship between the different data block such as* cis*-regulated mutations [17]. This work builds a hybrid model by combining both GWAS and eQTL models rather than a multiblock integration model. The data integration approach was suggested by utilizing multiple feature selection methods such as Principal Component Analysis (PCA), PLS, and LASSO [18]. They extracted the important factors using the dimensional reduction and feature selection methods and applied them on Cox survival models. However, combination effects of the multiblock data were ignored in this approach.

To tackle these limitations, we propose a novel Multiblock Discriminant Analysis (MultiDA) method for the integrative genomic study. The proposed method MultiDA makes the following main contributions.

- A new integrative genomic model for the discriminant analysis is introduced by exploiting class information.
- A sophisticated optimal solution is developed to solve the discriminant analysis problem in the integrative genomic model.

First, we built a novel integrative genomic model for the discriminant analysis. The class data is considered as one block, and the total squared correlation including the class block is maximized. The introduction of the class block to the multiblock model enables us to perform discriminant analysis in the integrative genomic model. Secondly, we propose a sophisticated method to solve the discriminant analysis problem in the new integrative genomic model. The discriminant analysis is essential in identifying biomarkers of human diseases in computational biology. Regardless, it has been overlooked in the multiblock analysis. The efficient algorithm for the discriminant analysis and assessment of its performance are explored in this paper.

## 2. Methods

### 2.1. Notation

**Figure 1:**
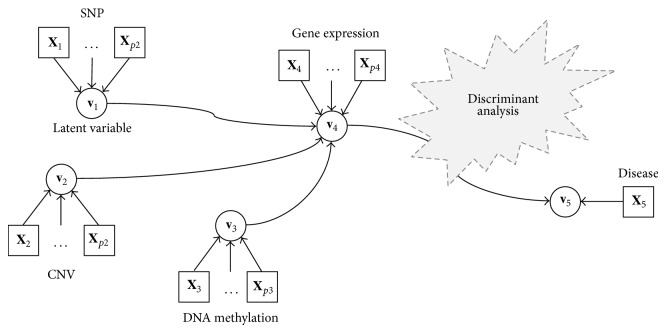
The conceptual graphic representation of the integrative genomic model. A rectangle represents a manipulated variable, and a circle represents a latent variable. The graphic representation illustrates the structure model that shows the relationship between SNP, CNV, DNA methylation, gene expression, and disease phenotype.

We suppose that there are *J* multiblock data. The multiblock data are measured on *N* numbers of the same set of observations. A block consists of a group of features that share common properties or represent one aspect of the sample. The multiblock data is denoted by **X** = {**X**_1_,…, **X**_*J*_}. The *j*th block data **X**_*j*_ is *P*_*j*_-dimensional zero mean column vectors **X**_*j*_ ∈ *ℜ* ^*N*×*P*_*j*_^. A matrix **C** = {*c*_*jk*_∣*c*_*jk*_ ∈ {0,1},  1 ≤ *j*,  *k* ≤ *J*} is a binary matrix that determines the linkage between the multiblock, where *c*_*jk*_ = 1 if the block *j* and the block *k* are connected or 0 if otherwise. In the proposed integrative genomic model, SNP, CNV, DM, GE, and class label (case or control) of the samples are considered as the multiblock components. For simplicity, **X**_1_, **X**_2_, **X**_3_, **X**_4_, and **X**_5_ represent SNP, CNV, DM, GE, and class label, respectively. Through this paper, we use *i* for the index of the sample and {*j*, *k*} for the multiblock. (*ı*) is used to denote a column vector of a matrix or an element of a vector. For instance, **X**_*i*__(*ı*)_ and *a*_*i*__(*ı*)_ represent the *ı*th column vector of the matrix **X**_*i*_ and the *ı*th element of the vector **a**_*i*_, respectively.  Figure 1 illustrates the conceptual overview of the multi-block data and framework.

### 2.2. Multiblock Discriminant Analysis

Multiblock Discriminant Analysis (MultiDA) builds a sparse association model by not only maximizing the total squared correlations between the multiblocks but also taking into account the discriminative factors in the model. MultiDA considers a linear subspace which is a construction of low-dimensional basis of the data. The linear subspaces of the multiblock, which maximize the total squared correlations, identify the significant factors of the association model with sparsity regularization. The linear subspace (or latent variable) **v**_*j*_ of the *j*th block is represented by

(1) $$\begin{array}{c} v_{j}=X_{j}\alpha_{j}, \end{array}$$

where **α**_*j*_ is a loading vector. Then, we introduce sparse regularization (elastic net penalization) on the loading vector to reduce the chance of including insignificant variables and to improve their interpretation. The sparse regularization has its advantage especially when the number of features is much larger than the sample number (*N* ≪ *P*_*j*_). Therefore, the basic objective function can be represented as

(2) arg⁡max⁡αj ∑j=1J ∑k=1,j≠kJcjkαj⊤Xj⊤Xkαkαj⊤Xj⊤Xkαkαj⊤Xj⊤Xjαjαk⊤Xk⊤Xkαks.t. αj⊤Xj⊤Xjαj=1, αj≤t1, αj2≤t2,j=1,…,J,

where |·| and ‖·‖^2^ represent *ℓ*_1_-norm and *ℓ*_2_-norm of the vectors, respectively, and *t*_1_ and *t*_2_ are the shrinkage parameters that determine the sparsity. Note that the basic objective function is equivalent to the Sparse Generalized Canonical Correlation Analysis (SGCCA) [17]. Since the integrative genomic model aims to represent gene expression regulated by the combinations of SNP, CNV, and DM, the matrix **C** can be defined as

(3) $$\begin{array}{c} C=\left[\begin{array}{ccccc} 0 & 0 & 0 & 1 & 0\\ 0 & 0 & 0 & 1 & 0\\ 0 & 0 & 0 & 1 & 0\\ 1 & 1 & 1 & 0 & 1\\ 0 & 0 & 0 & 1 & 0 \end{array}\right]. \end{array}$$

We further consolidate the model by (1) introducing a weight matrix of the correlation for the balance of the model and (2) providing discriminant analysis in the integrative genomic model. We also provide the sophisticated solution of the model while SGCCA heuristically estimates the optimal solution by following Wold's algorithm in the previous work [17].

#### 2.2.1. Weight Matrix for the Balance of the Model

The weight matrix of the correlation between the multiblocks, **d** = {*d*_*jk*_∣*d*_*jk*_ ∈ *ℜ*,  1 ≤ *j*,  *k* ≤ *J*}, is introduced in the model. In the original multiblock model, the correlation between gene expression and class label block tends to be overlooked. Instead, the sum of the squared pairwise correlations of **X**_1_, **X**_2_, **X**_3_, and **X**_4_ contributes large portions. The correlation weight matrix **D** gives an equal balance of the total squared correlations. In this paper, the weight matrix is defined as

(4) $$\begin{array}{c} D=\left[\begin{array}{ccccc} 0 & 0 & 0 & 1 & 0\\ 0 & 0 & 0 & 1 & 0\\ 0 & 0 & 0 & 1 & 0\\ 1 & 1 & 1 & 0 & 3\\ 0 & 0 & 0 & 3 & 0 \end{array}\right], \end{array}$$

where the correlation between gene expression and class label blocks is three times more weighted than others. Then, the matrix **D** simply replaces the matrix **C**.

#### 2.2.2. Discriminant Analysis

In the proposed integrative genomic model, we need to find discriminative genes that characterize diseases. However, the integrative genomic model is comprised of combinations of multiple linear regression models. Thus, discriminant analysis such as Logistic Regression (LR) and Linear Discriminant Analysis (LDA) cannot be embedded into the integrative genomic model. To solve this problem, we adapted the Discriminative Least Squares Regression (DLSR) method proposed by Xiang et al. [19]. DLSR was developed based on the linear regression model, and it is proved that DLSR provides equal or superior performance compared to other discriminant methods. The basic concept of DLSR is to enlarge the distance between classes by introducing slack variables. Whereas they considered a multi-class problem and developed its sparse version with *ℓ*_2,1_-norm regularization in their work, we reformulated its sparse method with elastic net penalization to suit our own needs. In DLSR, the slack variable is introduced into the ordinary linear regression problem:

(5) $$\begin{array}{c} Xa=y+b⊙m, \end{array}$$

where **y** is a dependent variable (*y*_*i*_ = {−1,1}, **y** ∈ *ℜ* ^*N*^), **X** is a multivariate independent variable (**X** ∈ *ℜ* ^*N*×*p*^), and **a** is a coefficient vector (**a** ∈ *ℜ* ^*p*^). **b** is a direction of the class, where its element *b*_*i*_ = −1 if *y*_*i*_ = −1 or 1 if otherwise (**b** ∈ *ℜ* ^*p*^). The Hadamard product operator ⊙ of the direction vector **b** and the slack variable vector **m** determines the distance between classes (**m** ∈ *ℜ* ^*p*^). The optimal solution will be covered in the next section.

#### 2.2.3. The Objective Function of MultiDA

We finally obtain the objective function of MultiDA:

(6) arg⁡max⁡αj ∑j=1J ∑k=1,j≠kJdjkαj⊤χj⊤χkαkαj⊤χj⊤χkαkαj⊤χj⊤χjαjαk⊤χk⊤χkαks.t. αj⊤χj⊤χjαj=1, αj≤t1, αj2≤t2,j=1,…,J,

where **χ**_*j*_ is defined as

(7) $$\begin{array}{c} \chi_{j}=\left\{\begin{array}{cc} X_{j}+b⊙m & \text{if}\;\;j=5\\ X_{j} & \text{if otherwise}. \end{array}\right. \end{array}$$

This setting enables one to perform discriminant analysis between gene expression and disease blocks.

### 2.3. Optimization

The optimal solution of (6) can be obtained by the Lagrangian function:

(8) L=−∑jJ ∑k=1,j≠kJdjkαj⊤χj⊤χkαkαj⊤χj⊤χkαk+∑jJzjαj⊤χj⊤χjαj−1+∑jJλjαj+∑jJ1−λj2αj2,

where *z*_*j*_ and *λ*_*j*_ are the Lagrangian multipliers. The Lagrangian function (8) is convex, although not differentiable. Therefore, the local optimum of (8) provides a global solution. The partial derivatives of the Lagrangian function with respect to **α**_*j*_ and *λ*_*j*_ are derived from

(9) ∂L∂αj−∑kJdjkαj⊤χj⊤χkαkχj⊤χkαk+zjχj⊤χjαj+λjsj+1−λjαj=0,

(10) ∂L∂λjαj⊤χj⊤χjαj−1=0,

where **s**_*j*_ is the vector of **a**_*j*_'s sign. Although the stationary equations have no closed form solutions, the optimal solution can be estimated by an iterative algorithm.

We can make (9) simple with the inner component:

(11) $$\begin{array}{c} \upsilon_{j}=\overset{J}{\underset{k,k\neq j}{\sum }}d_{jk}\left(\;\alpha_{j}^{⊤}\chi_{j}^{⊤}\chi_{k}\alpha_{k}\;\right)\chi_{k}\alpha_{k}. \end{array}$$

Then, by introducing the inner component **υ**_*j*_ into (9), the solution of **α**_*j*_ can be written as

(12) $$\begin{array}{c} \alpha_{j}={\left[\;z_{j}\left(\;\chi_{j}^{⊤}\chi_{j}+\frac{1-\lambda_{j}}{z_{j}}\;\right)\;\right]}^{-1}\left(\;\chi_{j}^{⊤}\upsilon_{j}-\lambda_{j}s_{j}\;\right). \end{array}$$

In (11), (**α**_*j*_^*⊤*^ **χ**_*j*_^*⊤*^ **χ**_*k*_ **α**_*k*_) is a squared correlation between the latent variables of the *i*th and *j*th block, which is a scalar. Therefore, the inner component is computed by **α**_*j*_ of the previous iteration, and then new **α**_*j*_ is updated in iterations.

Equation (12) is the normal equation of the regression of **υ**_*j*_ on **χ**_*j*_ with ridge and shrinkage parameter [20]. The final solution can be obtained by using the Univariate Soft-Thresholding (UST) method [21]:

(13) $$\begin{array}{c} \alpha_{j_{\left(ı\right)}}=\mathrm{sign}\left(\;\chi_{j_{\left(ı\right)}}^{⊤}\upsilon_{j}\;\right){\left(\;\left|\;\chi_{j_{\left(ı\right)}}^{⊤}\upsilon_{j}\;\right|-\lambda_{j}\;\right)}_{+}, \end{array}$$

where sign⁡(*x*) returns a sign of *x*, that is, 1 if *x* ≥ 0 or −1 if otherwise. (*x*)_+_ returns only positive values of *x* (i.e., *x* if *x* ≥ 0 or 0 if otherwise). *λ*_*j*_ can be obtained by *K*-fold cross-validation that minimizes mean squared errors. The parameter *z*_*j*_ can be ignored because the solution of **α**_*j*_ is normalized by (10):

(14) $$\begin{array}{c} \alpha_{j}=\frac{\sqrt{N}\alpha_{j}}{\left\|\chi_{j}\alpha_{j}\right\|}. \end{array}$$

For the discriminant analysis between gene expression and disease data blocks, the optimum of the slack variable **m** and the loading vector **α**_4_ can be estimated by solving the following optimization problem:

(15) arg⁡max⁡α4,m 12χ4α4−υ5+b⊙m2s.t. α4≤ξ1, α42≤ξ2.

The Lagrangian function of (15) is *ℒ* = (1/2)‖**χ**_4_ **α**_4_ − **υ**_5_ − **b**⊙**m**‖^2^ + *λ*_4_ | **α**_4_ | +((1 − *λ*_4_)/2)‖**α**_4_‖^2^. The derivative of the Lagrangian function with respect to **α**_4_ is

(16) $$\begin{array}{c} \frac{L}{\partial \alpha_{4}}=\chi_{4}^{⊤}\chi_{4}\alpha_{4}-\chi_{4}^{⊤}\gamma +\lambda_{4}s+\left(\;1-\lambda_{4}\;\right)\alpha_{4}=0, \end{array}$$

where **s** is the sign of **α**_4_ and **γ** = **υ**_5_ + **b**⊙**m**. Thus, the equation of **α**_4_ becomes

(17) $$\begin{array}{c} \alpha_{4}={\left(\;\chi_{4}^{⊤}\chi_{4}+1-\lambda_{4}\;\right)}^{-1}\left(\;\chi_{4}^{⊤}\left(\;\gamma \;\right)-\lambda_{4}s\;\right). \end{array}$$

Finally, the optimal solution of **α**_4_ for the discriminative analysis is

(18) $$\begin{array}{c} \alpha_{4_{\left(ı\right)}}=\mathrm{sign}\left(\;\chi_{4_{\left(ı\right)}}^{⊤}\gamma \;\right){\left(\;\left|\;\chi_{4_{\left(ı\right)}}^{⊤}\gamma \;\right|-\lambda_{4}\;\right)}_{+}. \end{array}$$

*λ*_4_ is also determined by *K*-fold cross-validation that minimizes mean squared errors like other *λ*_*j*_'s. The optimal solutions of **m** are simply derived from [19]

(19) $$\begin{array}{c} m=\max\left(\;b⊙\left(\;\chi_{4}\alpha_{4}-\upsilon_{5}\;\right),0\;\right). \end{array}$$

The brief algorithm is described in Algorithm 1. In the algorithm, *r* represents a rank of the subspace, which determines the dimension of the subspace. For instance, **α**_*j*_^*r*^ is *r*th rank of **α**_*j*_. MultiDA optimizes the first rank subspace and iterates the optimization until the multiblock has no information. In lines 10–14 of Algorithm 1, Wold's procedure guarantees the convergence [22].

## 3. Experiment Results

The goal of the assessment is to identify significant factors of the integrative genomic model with the multiblock data, specifically the discriminative factors of human disease. The discriminant factors include disease-specific locations or regions of SNP, CNV, DNA methylation, and gene expression against normal patients.

### 3.1. Simulation Study

We assessed the performance of the proposed method MultiDA through simulated data. Simulation data of various complexities were considered. Generation's schemes of the simulation data for the assessment were extended from the previous related works [16, 23].

Four generation functions of different complexity are defined as shown in Table 1. Type_1_(*μ*) generates *p*-dimensional normally distributed random variables of a given mean (*μ*) and a variance (**I**_*p*×*p*_), where **I**_*p*×*p*_ is an *p* × *p* identity matrix. Type_2_(*μ*, *δ*) generates more complicated data than Type_1_(*μ*). In Type_2_(*μ*, *δ*), a random model with a threshold (*δ*) is implemented with the function 1_*δ*_. Given a uniform distributed random value (*u*), 1_*δ*_ = 1 if *u* ≤ *δ* or 0 if otherwise. Type_3_(*μ*, *ρ*) considers multicollinearity data in which more than two variables are highly correlated. The matrix data are generated by multivariate normal distribution *𝒩*(*μ*, Σ_*p*×*p*_). The covariance structure Σ_*p*×*p*_ is built by the first order of autoregressive process. Type_4_(*μ*, *σ*) generates *p*-dimensional normally distributed random variables from a given mean (*μ*) and a variance (*σ*).

The first three multiblocks (**X**_*j*_ ∈ *ℜ* ^*N*×*P*_*j*_^, 1 ≤ *j* ≤ 3) were simulated by compounding the generation functions as defined in Table 2, where *P*_1_ = 100, *P*_2_ = 200, *P*_3_ = 300, and *N* = 500. For instance, the first five columns of **X**_1_ were generated by Type_1_(2.4) and the following five columns were by Type_1_(−2.6). The next 30 columns were generated by the generation model with a threshold Type_2_(1,0.6). The remaining columns of **X**_1_ were generated by the multicollinearity random variables Type_3_(0,0.8). Then, we considered the multiblock linear model, **X**_4_ = ∑_*j*=1_^3^ **X**_*j*_ **B**_*j*_ + Ξ, where **B**_*j*_ is a *P*_*j*_ × *P*_4_ loading matrix and Ξ is a *P*_*j*_ × *P*_4_ dimensional normally distributed noise matrix (*P*_4_ = 50). We assumed that only the first ten variables of each block are significant to explain **X**_4_. The fifth block **X**_5_ is class label block. Given a coefficient vector **B**_4_ ∈ *ℜ* ^*P*_4_×1^ (all zeros but the first ten), the probability of disease *π* was computed by using

(20) $$\begin{array}{c} \pi =\frac{\exp\left(X_{4}B_{4}\right)}{1+\exp\left(X_{4}B_{4}\right)}. \end{array}$$

Then, the binary class label block was generated using the Bernoulli distribution with the probability *π*.

The simulation study was examined with 50 replications to assess the reproducibility. We compared the performance of MultiDA with the related methods, Sparse Canonical Correlation Analysis (SCCA) [24] and Sparse Generalized Canonical Correlation Analysis (SGCCA) [17]. SCCA is a two-block method that maximizes the correlation between independent *𝒳* and response variable *𝒴*. In SCCA, the three blocks of data were combined into a single block (*𝒳* = {**X**_1_, **X**_2_, **X**_3_}), and the block GE was considered as response (*𝒴* = **X**_4_). The class label block was not considered in SCCA. The multiblock method SGCCA was tuned to be compatible with the proposed integrative genomic model. Note that the same matrix **C** was used in SGCCA, but SGCCA did not take the discriminant analysis into account.

**Figure 2:**
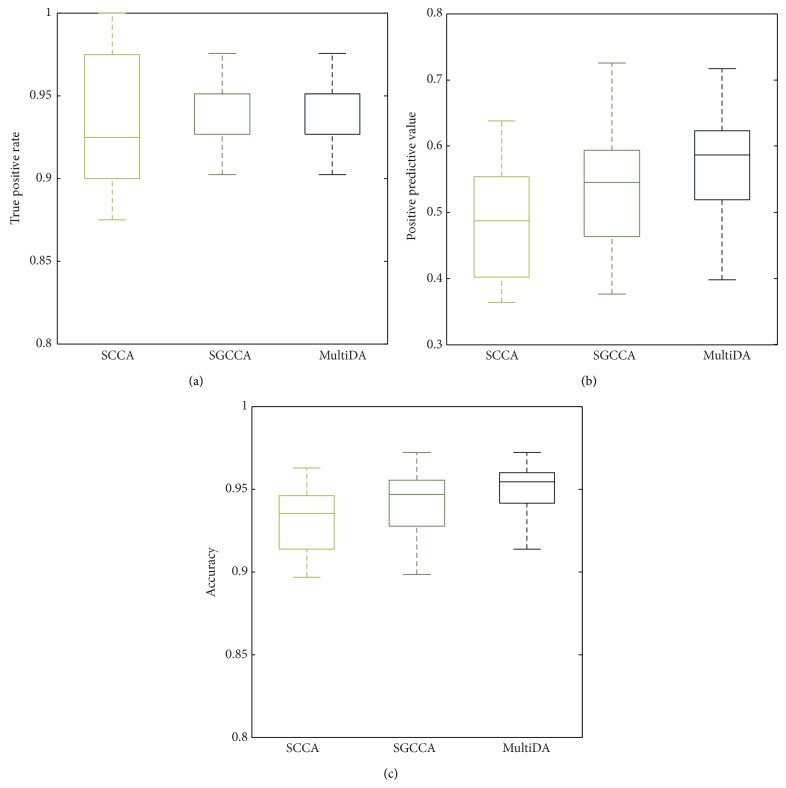
Performance comparison in simulation study: (a) True Positive Rate; (b) Positive Predictive Value; (c) Accuracy.

We examined the performance by how well they correctly identify significant factors of the integrative association model. Given a ground truth, we computed a confusion matrix and measured True Positive Rate (TPR), Positive Predictive Value (PPV), and Accuracy (ACCU). In the sparse setting, the true negatives are relatively much larger than false positives. Therefore, True Negative Rates (TNR) and Negative Predictive Values (NPV) were not included in this paper. The results of the simulation experiment are illustrated in Figure 2. The proposed method MultiDA (0.93 ± 0.03) and the multiblock method SGCCA (0.93 ± 0.03) outperformed SCCA (0.83 ± 0.24) in terms of TPR. It supports that the multiblock methods reduce false negatives that incorrectly identify the significant as the insignificant. MultiDA appeared as the best performance in PPV and ACCU. MultiDA produced 0.58 ± 0.07 and 0.95 ± 0.01 for PPV and ACCU, respectively. Higher PPV values represent lower false positives that incorrectly identify the insignificant as the significant. The PPV and ACCU of SCCA were 0.48 ± 0.15 and 0.89 ± 0.14 and were 0.54 ± 0.08 and 0.94 ± 0.01 for SGCCA, respectively.

### 3.2. Human Brain Data of Schizophrenia

Human brain data were obtained from three major psychiatric disorders such as schizophrenia (SZ), bipolar disorder (BP), and major depression (DP) as well as from control group. Specifically, 39 samples of SZ, 35 samples of BP, 12 samples of DP, and 43 samples of control were provided from the Stanley Medical Research Institute. SNP, CNV, DNA methylation, and gene expression data were acquired from the human prefrontal cortex of the 129 samples in the preparation of this experiment. For each individual, 10,760 SNPs after removing highly correlated ones, 1,028 CNVs, 20,769 DNA methylations, and 19,767 gene expressions were examined. Due to the recent research that reported that genetic effects may be largely shared in major psychiatric disorders such as autism spectrum disorder, attention deficit-hyperactivity disorder, bipolar disorder, major depressive disorder, and schizophrenia, we considered those psychiatric diseases together and performed MultiDA to identify discriminate factors against the control [25, 26].

The multiblock data was analyzed by MultiDA. As a result of the analysis, 78 SNPs, 30 CNVs, 47 DNA methylations, and 35 genes were detected, where the high correlation between the connections was found. The potential gene markers of the psychiatric disorders were inferred from the result of the proposed method. The genes physically located near the selected SNPs and the genes corresponding to the result of CNV and the DNA methylation were chosen. Significantly observed genes among the results of MultiDA are listed in Table 3, where the data source of the gene and literature regarding the psychiatric disorders are described.

**Figure 3:**
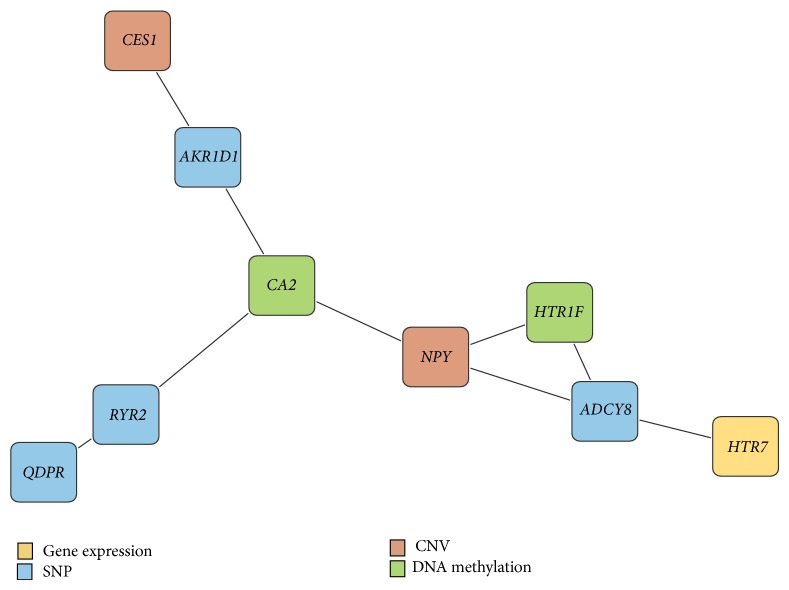
The gene regulatory network searched with the gene results by STRING database. The legend shows the data source of the gene.

The gene regulatory network of the genes from the result was searched by STRING database [27]. Among a number of the retrieved interactions, we take note of one gene regulatory network illustrated in Figure 3. The interaction network consists of* HTR7*,* ADCY8*,* HTR1F*,* NPY*,* CA2*,* RYR2*,* QDPR*,* AKR1D1*, and* CES1* gene.* HTR7* is inferred from the gene expression set,* HTR1F* and* CA2* are from the DNA methylation expression,* NPY* and* CES1* are from the CNV, and the others are from the SNP data. The negative coefficient of* HTR1F* in the model may support the widely accepted notion that DNA methylation suppresses gene regulation impeding the binding of transcriptional proteins to the gene [34]. In particular, the* HTR7* gene (5-hydroxytryptamine receptor 7) is a major neurotransmitter in the central nervous system, and a number of literatures related to bipolar and schizophrenia disorder are reported [28]. Interestingly, the* HTR7* gene was found in the gene expression data block in this study, while the other previous researches reported the gene with GWAS on the SNP data block. The gene may have strong incorporated interactions with other heterogeneous data, which is consequently considered to be significant in the integrative model. It supports the strength of the integrative approach. Moreover, we found that* HTR7* and* NPY* are in the same pathway, which is* neuroactive ligand-receptor interaction*, where the* NPY* gene is also a neurotransmitter in the brain and is known to play an important role in the emotional process [30]. A large number of psychiatric disorder susceptible genes were associated with this pathway [25].* ADCY8*, which interacts with both* HTR7* and* NPY*, may be potentially a susceptibility gene that causes the psychiatric disorders. In previous research [35], they found that* ADCY8* is a susceptibility gene for avoidance behavior on mouse and also found that it indirectly induces the susceptibility on human mood disorders. Our result supports their claim.

## 4. Conclusion

In this paper, we developed the novel Multiblock Discriminant Analysis method in order to dissect the mechanism of complex human disease using multiple genetic data. The genomic association study with single type data may fall short of identifying the mechanisms of the diseases. On the other hand, MultiDA enables comprehensive analysis using multiple genetic data. Moreover, MultiDA provides analysis for the special setting of binary class data, where it greatly detects discriminative factors in the integrative genomic model. The simulation experiments support the outstanding performance of the proposed methods. As a target application, psychiatric disorder disease data, including SNP, CNV, DNA methylation, and gene expression, were analyzed in the integrative genomic model. Among the large number of variables of each block, candidate biomarkers were proposed as significant components of the disease mechanism. The proposed methods capture the global profile of the mechanism that conventional single or two block methods fail to detect. This promising tool for the integrative genomic study can provide flexible extensibility for new types of data in the era, superseding new high-throughput technologies.

## Figures and Tables

**Algorithm 1:**
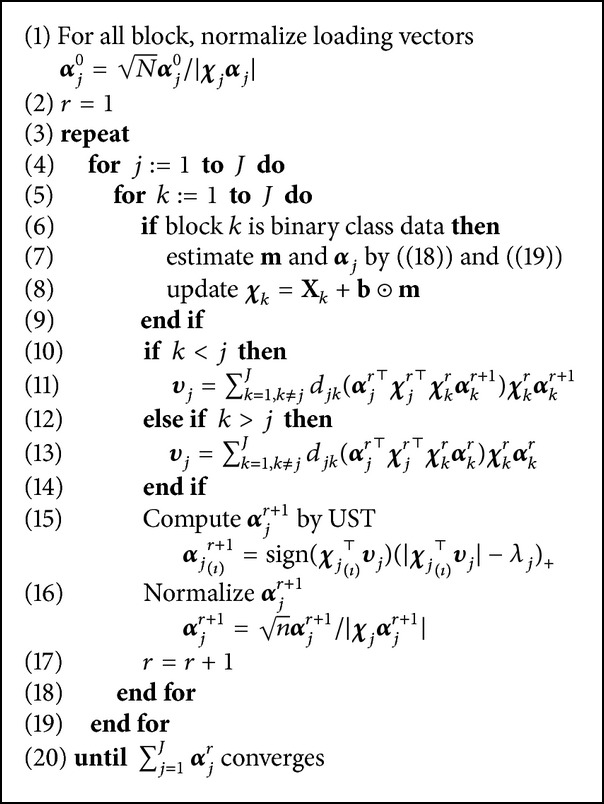
Discriminant multiblock analysis.

**Table 1:** Generation functions.

Function	Model
Type_1_(*μ*)	**x** = *μ* + *ϵ*, *ϵ* ~ *𝒩*(0, **I**)
Type_2_(*μ*, *δ*)	**x** = *μ* + 1_*δ*_ + *ϵ*, *ϵ* ~ *𝒩*(0, **I**)
Type_3_(*μ*, *ρ*)	**x** ~ *𝒩*(*μ*, Σ_*p*×*p*_)
Type_4_(*μ*, *σ*)	**x** ~ *𝒩*(*μ*, *σ * **I**_*p*×*p*_)

**Table 2:** Scheme of the simulation data.

Simulation data	Generation model type	Column index
**X**_1_	**x**_*i*_ = Type_1_(2.4)	1 ≤ *ı* ≤ 5
	**x**_*i*_ = Type_1_(−2.6)	6 ≤ *ı* ≤ 10
	**x**_*i*_ = Type_2_(1,0.6)	11 ≤ *ı* ≤ 40
	**x**_*i*_ = Type_3_(0,0.8)	41 ≤ *ı* ≤ 100

**X**_2_	**x**_*i*_ = Type_1_(3)	1 ≤ *ı* ≤ 5
	**x**_*i*_ = Type_1_(4)	6 ≤ *ı* ≤ 10
	**x**_*i*_ = Type_3_(0,0.9)	11 ≤ *ı* ≤ 60
	**x**_*i*_ = Type_4_(2,2)	61 ≤ *ı* ≤ 200

**X**_3_	**x**_*i*_ = Type_1_(5)	1 ≤ *ı* ≤ 5
	**x**_*i*_ = Type_1_(−3)	6 ≤ *ı* ≤ 10
	**x**_*i*_ = Type_4_(0,1)	11 ≤ *ı* ≤ 210
	**x**_*i*_ = Type_3_(0,0.9)	211 ≤ *ı* ≤ 300

**Table 3:** The gene results from MultiDA with psychiatric disorders.

Gene	Chromosome	Location	Source	ID	MAF	Reference
HTR7	10	10q21-q24	GE	7934970		[28]
APOE	19	19q13.2	DM	cg14123992		[29]
TRPM1	15	15q13.3	DM	cg18085517		
EPHB1	3	3q21-q23	CNV	CNP12652		
NPY	7	7p15.1	CNV	CNP2267		[30]
QKI	6	6q26	SNP	rs1336225	0.18	
SLC15A1	13	13q32.3	SNP	rs9517421	0.17	[31]
NPAS3	14	14q13.1	SNP	rs1124910	0.25	[32]
C15orf53	15	15q14	SNP	rs1433876	0.29	[33]
